# Cytocompatibility of Titanium, Zirconia and Modified PEEK after Surface Treatment Using UV Light or Non-Thermal Plasma

**DOI:** 10.3390/ijms20225596

**Published:** 2019-11-08

**Authors:** Linna Guo, Ralf Smeets, Lan Kluwe, Philip Hartjen, Mike Barbeck, Claudio Cacaci, Martin Gosau, Anders Henningsen

**Affiliations:** 1 Department of Oral and Maxillofacial Surgery, University Hospital Hamburg-Eppendorf, 20246 Hamburg, Germany; guolinna001@gmail.com (L.G.); r.smeets@uke.de (R.S.); kluwe@uke.de (L.K.); p.hartjen@uke.de (P.H.); m.barbeck@uke.de (M.B.); m.gosau@uke.de (M.G.); 2 Department of Oral and Maxillofacial Surgery, University Hospital Hamburg-Eppendorf, 20246 Hamburg, Germany; 3 Department of Neurology; University Hospital Hamburg-Eppendorf, 20246 Hamburg, Germany; 4 Implant Competence Centrum, Weinstr. 4, 80333 Munich, Germany; dr.cacaci@me.com

**Keywords:** ultraviolet light, non-thermal plasma, modified polyetheretherketone, titanium, zirconia, dental abutments

## Abstract

A number of modifications have been developed in order to enhance surface cytocompatibility for prosthetic support of dental implants. Among them, ultraviolet (UV) light and non-thermal plasma (NTP) treatment are promising methods. The objective of this study was to compare the effects of UV light and NTP on machined titanium, zirconia and modified polyetheretherketone (PEEK, BioHPP) surfaces in vitro. Machined samples of titanium, zirconia and BioHPP were treated by UV light and NTP of argon or oxygen for 12 min each. Non-treated disks were set as controls. A mouse fibroblast and a human gingival fibroblast cell line were used for in vitro experiments. After 2, 24 and 48 h of incubation, the attachment, viability and cytotoxicity of cells on surfaces were assessed. Results: Titanium, zirconia and BioHPP surfaces treated by UV light and oxygen plasma were more favorable to the early attachment of soft-tissue cells than non-treated surfaces, and the number of cells on those treated surfaces was significantly increased after 2, 24 and 48 h of incubation (*p* < 0.05). However, the effects of argon plasma treatment on the cytocompatibility of soft tissue cells varied with the type of cells and the treated material. UV light and oxygen plasma treatments may improve the attachment of fibroblast cells on machined titanium, zirconia and PEEK surfaces, that are materials for prosthetic support of dental implants.

## 1. Introduction

Dental implants have become a safe and reliable treatment method in patients with reduced dentition [1,2]. The long-term survival and success of dental implants is influenced by various factors [3,4,5]. Among others, soft-tissue adhesion on transmucosal parts of an implant (abutments) plays a key role in the long-term success of dental implants. A good soft-tissue sealing can contribute to reduced bacterial colonization and reduced risk of inflammation [6]. In order to achieve soft-tissue sealing, human gingival fibroblasts (HGFs), which are regarded as the main cell type in peri-implant soft tissues [7], need to adhere to the surface before bacteria, so that a cellular layer can cover the abutment, making the surface less available for bacterial attachment [8,9]. Abutments as transmucosal parts of an implant are expected to achieve early soft-tissue adhesion, demonstrating good soft-tissue biocompatibility. Modifications of implant abutment surfaces have been the focus of recent research [10,11,12].

Modifications of the biomaterial surface topography, and chemical modifications, have been introduced to optimize commonly used implant abutment systems in recent years [13]. Most of those modifications are applied during manufacturing, but most dental implants are transported in customary packages that are permeable to air. It was reported that, after 4 weeks of storage in customary available blisters, the surfaces of titanium and zirconia are saturated with carbon compounds [14]. Suzuki et al. found that, after 2 weeks of healing in a rat model, the strength of osseointegration on 4-week-aged titanium surfaces was reduced by 50%, compared to newly prepared titanium implants [15]. In previous studies, micro-rough titanium and zirconia surfaces were treated by ultraviolet (UV) light or non-thermal plasma (NTP) in a short time, and the results showed that either method was able to significantly increase the wettability and oxygen content of the surfaces, and decrease the amount of carbon remnants [16,17,18]. Furthermore, the results also indicated that NTP and UV light led to significantly improved cell attachment of murine osteoblasts on both materials. Due to the manageable size of the required equipment, and the feasible processing times, these methods could be easily integrated into the daily routine of a dental practice. UV light and NTP can be considered promising approaches to improve the biocompatibility of dental implant materials [19,20,21,22]. 

Titanium, with excellent biocompatibility and mechanical properties, is most often used as abutment material for dental implants. However, due to its dark-grayish metallic color, the application of titanium abutments in the esthetic zone is limited [23]. Furthermore, some studies indicate that titanium alloys may contain an allergenic potential [24]. Thus, alternative materials have been introduced, e.g., zirconia, which may be recommendable in esthetically challenging areas but may also be prone to fractures [25]. Recently, high performance polymer materials, e.g., polyetheretherketone (PEEK), have gained attention, due to their favorable mechanical and biocompatible properties [26]. BioHPP, formed of PEEK containing 25% ceramic fillers, may also be a promising abutment material for dental implants [27,28]. Further advantages of this material may be its low weight, color stability, similar elasticity to bone, low allergenic potential, low plaque accumulation and lack of corrosion [29]. However, like titanium and zirconia as materials for abutments, little is known about the bioactivity of PEEK over time during storage. Therefore, whether surface treatment of these materials using UV light or NTP may improve the cytocompatibility of soft-tissue cells remains an interesting approach, that should be investigated further.

The present study aimed to assess the effects of UV irradiation and NTP treatment on interactions between the polished surfaces of three types of abutment materials (titanium, zirconia and BioHPP) and soft tissue cells (mouse fibroblast cell line L929 and HGFs), concerning cell attachment, morphology, cell viability and cytotoxicity.

## 2. Results

### 2.1. Cell Attachment and Morphology

**Figure 1:**
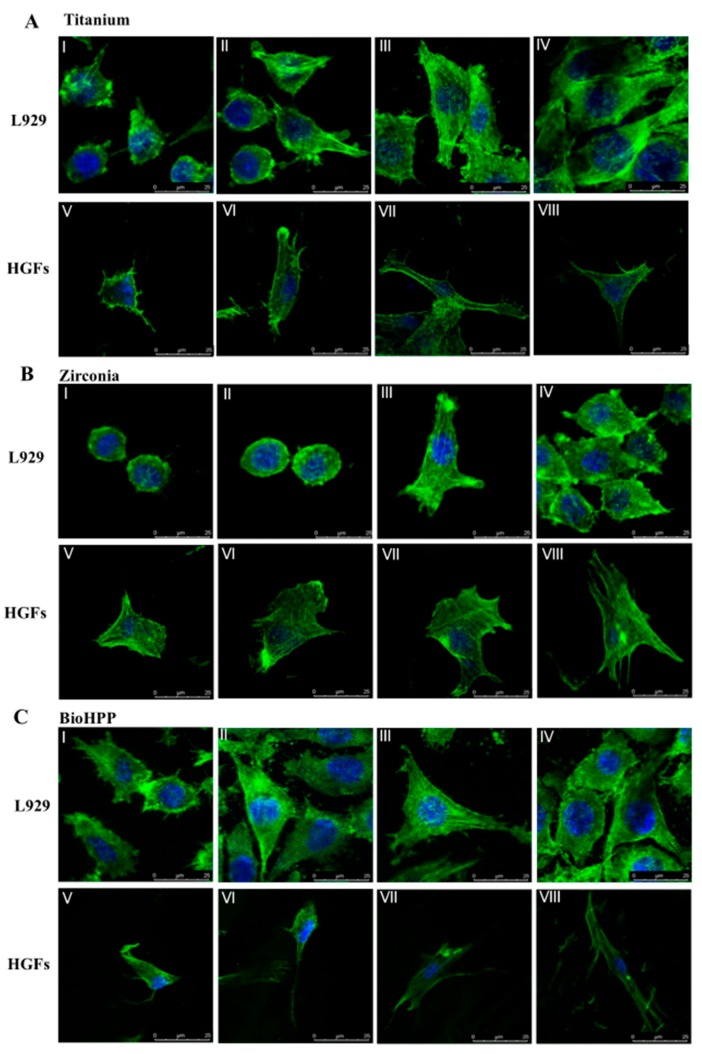
Cell attachment and morphology on different, surface-treated titanium, zirconia and BioHPP after 24 h. Representative examples of cytoskeleton stained with phalloidin after 24 h of incubation on controls (**I**,**V**), argon plasma treated (**II**,**VI**), oxygen plasma treated (**III**,**VII**) and UV light treated (**IV**,**VIII**) surfaces of titanium (**A**), zirconia (**B**) and BioHPP (**C**) using confocal microscopy. Both L929 and human gingival fibroblasts (HGFs) increased in volume and adhered flatly to the surfaces. Morphology of cells was more extended and cells were larger, especially on oxygen plasma and UV light treated surfaces. In the oxygen plasma and UV light treated groups, L929 and HGFs extended most, and had more filopodia-like cell processes.

**Figure 2:**
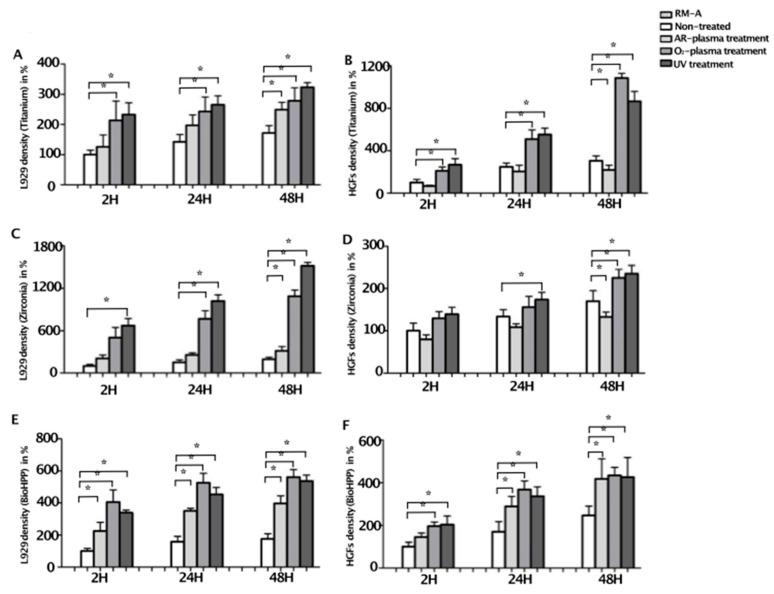
The number of living cells attached to different surface-treated titanium, zirconia and BioHPP, after 2, 24 and 48 h. The number of living cells attached to controls and surface treated titanium (**A**,**B**), zirconia (**C**,**D**) and BioHPP (**E**,**F**) was counted after 2, 24 and 48 h of incubation using live–dead staining. After 2 h of incubation, only a small number of cells adhered to the surfaces of the three materials in the control groups. During 48 h of incubation, the number of cells increased steadily. Generally, compared to controls, a significantly larger number of cells attached after oxygen plasma of UV light treatment on all disks and, additionally, after argon plasma treatment on BioHPP disks. For better visualization, results are shown in percent of 2 h negative controls ± standard deviation, * *p* < 0.05.

Generally, compared to controls, cells on surfaces treated by UV light and oxygen plasma were larger and more elongated (Figure 1), and, after 2, 24 and 48 h of incubation following live–dead staining, the number of cells attached were significantly higher after these treatments (Figure 2). 

**Figure A1:**
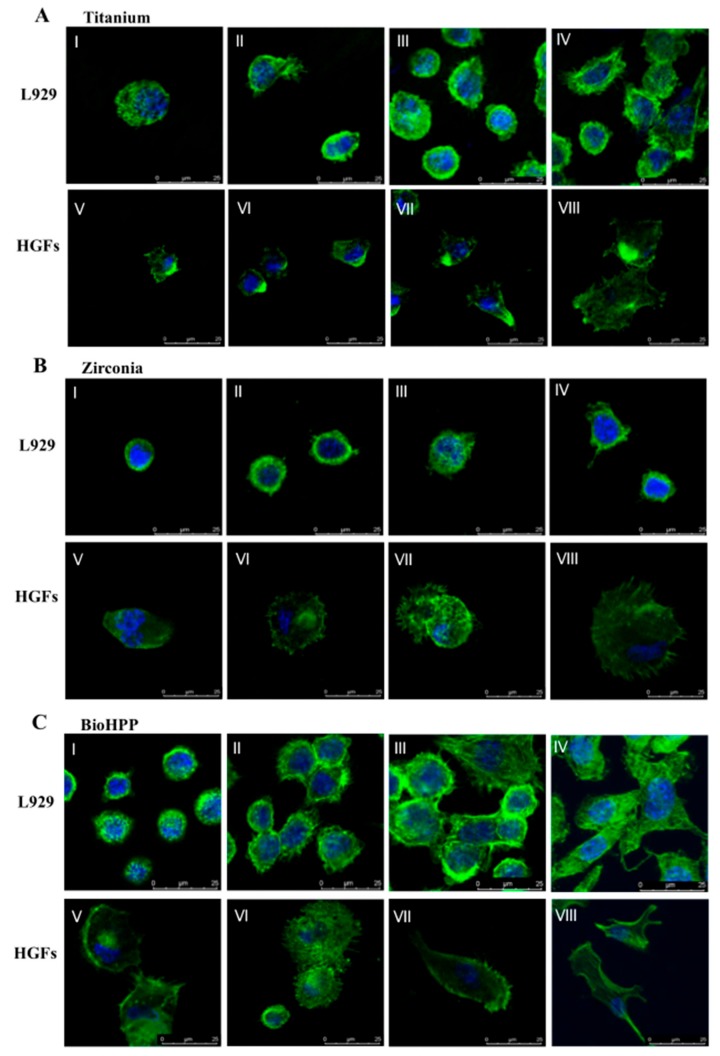
Cell attachment and morphology on different surface-treated titanium, zirconia and BioHPP after 2 h.

After 2 h of incubation, a small number of cells adhered to the surfaces of the three materials in the control group and cells showed very rare filopodia-like cell processes. Compared to controls, a larger number of cells attached on argon plasma treated surfaces, demonstrating more filopodia-like cell processes. However, on oxygen plasma- or UV light-treated surfaces of the three materials, a significantly increased number of cells attached (Figure 2). Adherent cells were larger, extended and showed a larger number of filopodia-like cell processes, compared to controls and argon plasma treated samples (Figure A1). 

**Figure A2:**
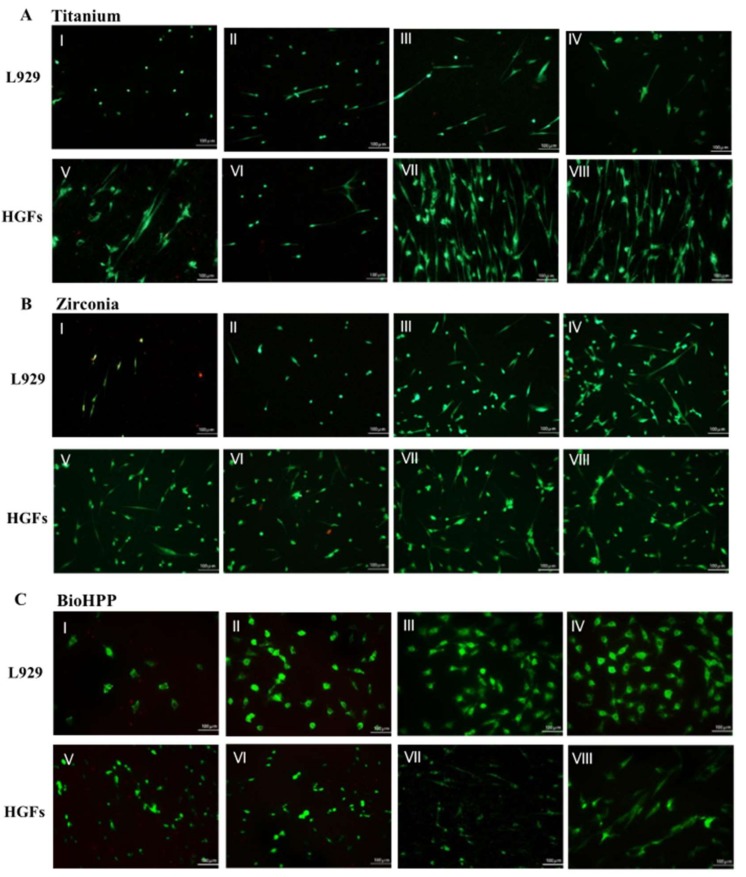
Cell attachment and morphology on different surface-treated titanium, zirconia and BioHPP after 48 h.

After 24 h of incubation, both L929 and HGFs increased in all groups. Compared to controls, a significantly increased number of cells attached after UV light and oxygen plasma treatment on titanium (*p* < 0.05), as well as a significantly increased number of L929 on oxygen plasma and UV light treated zirconia (*p* < 0.01), and a significantly increased number of HGFs on UV light-treated zirconia (*p* < 0.05, Figure 2). On BioHPP, most cells attached after oxygen plasma treatment, but numbers of cells after UV light and argon plasma treatment were also increased compared to controls (Figure 2). Morphology of cells was more extended and cells were larger, especially on oxygen plasma- and UV light-treated surfaces (Figure 1). In the oxygen plasma- and UV light-treated groups, L929 and HGFs extended and formed a long and fusiform shape. After 48 h, the trend was continuing and the number of cells on UV light- and oxygen plasma-treated titanium and zirconia samples were significantly increased, as well as on NTP- and UV light-treated BioHPP samples, compared to controls (*p* < 0.05, Figure 2 and Figure A2). However, the number of HGFs on argon plasma-treated titanium and zirconia was significantly decreased (*p* < 0.05, Figure 2).

### 2.2. Viability 

**Figure 3:**
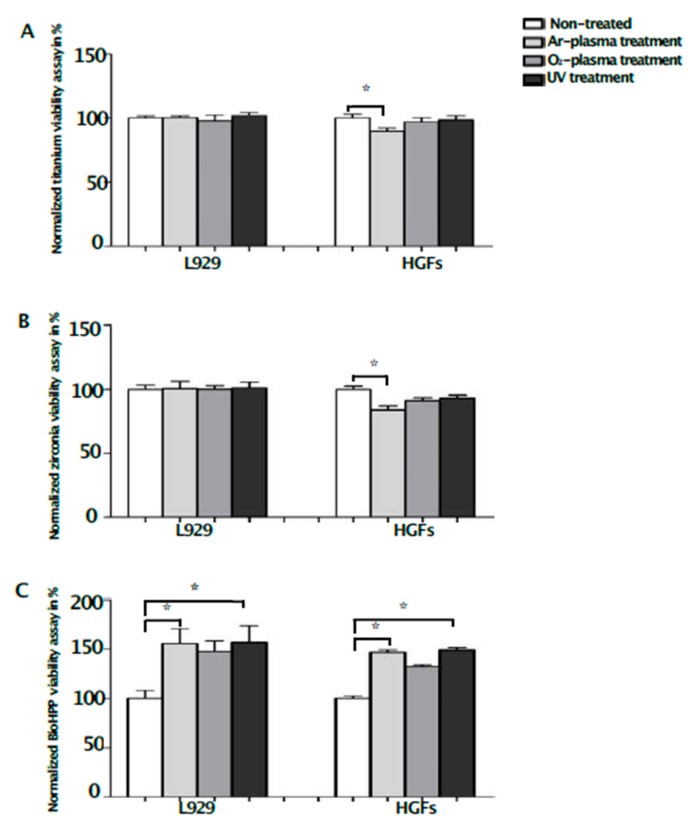
Viability of L929 and HGFs on different surface-treated titanium, zirconia and BioHPP after 48 h. Viability of L929 and HGFs on controls and surface-treated titanium (**A**), zirconia (**B**) and BioHPP (**C**) disks after 48 h of incubation. While the viability of HGFs on titanium and zirconia was significantly lower after argon plasma treatment, the viability of both used cell lines was significantly increased on BioHPP (* *p* < 0.05) after argon plasma and UV light treatment, compared to controls. However, the viability of both cell lines was also increased on oxygen plasma-treated BioHPP disks, but differences were not significant.

Whereas any surface treatment showed only minimal effects on titanium and zirconia disks regarding viability, UV light, as well as NTP, increased the viability of L929 and HGFs on BioHPP (Figure 3). The differences were significant after argon plasma and UV light treatment, compared to controls (*p* < 0.05), without significant differences between the treatments. Compared to controls, the viability of HGFs on zirconia (84% ± 3.12%) and titanium (90% ± 2.60%) disks was significantly lower after argon plasma treatment (Figure 3A,B), confirming the differences regarding cell attachment.

### 2.3. Toxicity

**Figure 4:**
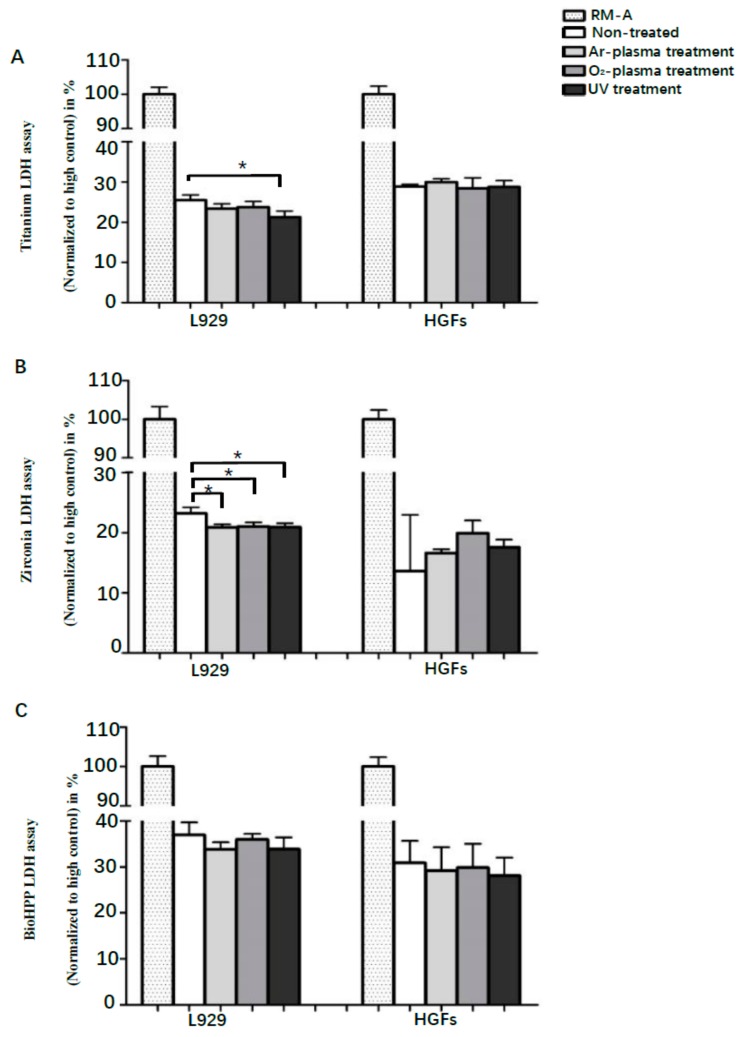
Toxicity of L929 and HGFs on different surface-treated titanium, zirconia and BioHPP after 48 h. Toxicity of L929 and HGFs on controls and surface-treated titanium (**A**), zirconia (**B**) and BioHPP (**C**) disks after 48 h of incubation. UV light treatment led to significantly decreased toxicity on titanium and zirconia, and NTP on zirconia, using L929 cells. However, there were no significant differences on titanium, zirconia and BioHPP using HGFs.

Cytotoxicity was assessed by LDH assay after 48 h of incubation, and compared to RM-A samples. Although any surface treatment led to decreased toxicity of titanium, zirconia and BioHPP disks using L929 cells, differences were only significant after UV light treatment on titanium (*p* < 0.05), and after any surface treatment on zirconia disks (*p* < 0.05), without significant differences between the treatments (Figure 4). Generally, differences between controls and experimental groups were not significant using HGFs. Contrary to the results using L929, the toxicity of zirconia disks was marginally increased after any surface treatment using HGFs, but differences were not significant. However, results in the zirconia HGFs control group showed a high standard deviation. 

## 3. Discussion

Titanium, zirconia and PEEK are the most frequently used materials for implant abutments. To the knowledge of the authors, no previous study has compared the effects of UV light and NTP surface treatments of these materials, in order to evaluate their effects on soft-tissue cytocompatibility. L929 cells and HGFs were used to assess cell attachment, morphology, viability and toxicity on titanium, zirconia and BioHPP (ceramic reinforced polyetheretherketone) samples, after 12 min of treatment using UV light, NTP of argon, or oxygen, compared to non-treated controls. The results of the present study suggest that UV light- and oxygen plasma-treated titanium, zirconia and BioHPP surfaces may be more conducive to the early attachment of soft-tissue cells. Compared to non-treated surfaces, the cytotoxicity of L929 cells was significantly decreased on the UV light-treated surfaces of titanium and zirconia and NTP-treated surfaces of zirconia. Consequently, argon plasma-treatment could improve the attachment and viability of L929 and HGFs on BioHPP surfaces, but decrease the attachment and viability of HGFs on titanium and zirconia surfaces. 

HGFs, which are one of the most commonly used fibroblast cell lines, were used as one of the models in this study. It is reported that HGFs are the most abundant cells in the gingiva, which can produce an extracellular matrix for wound healing and regeneration after a dental implant placement [30]. Effective attachment of HGFs on implant material surfaces plays a key role in soft-tissue integration [31]. However, a monolayer culture in vitro may lead to rearrangements of the cytoskeleton and limited cell–cell contact [32]. The murine fibroblast cell line L929 was used to increase the reliability of the results. Both cell lines offer reliable in vitro models, with similarities to primary human fibroblast cell lines, but, since they are not able to mimic biological processes in total, the significance of this study is limited.

Although titanium and zirconia are most frequently used as abutment materials for dental implants, BioHPP, as a modified PEEK strengthened by ceramic filler material, is regarded as a promising material, due to its excellent material performances [33,34]. From the perspective of mechanical properties, BioHPP possesses a level of elasticity which is comparable to human spongious bone, which may be favorable for implant-supported prosthetic solutions [27]. In this study, all samples were polished in order to mimic the trans-gingival surfaces of commercially available titanium, zirconia and BioHPP abutments. The work of Hersel et al. reported that attachment, spreading, cytoskeleton development and the formation of cell-matrix adhesions are a cascade of events that take place during cell adhesion onto the surface of biomaterial [35]. During cell spreading and focal adhesion formation, the survival and proliferation of anchorage-dependent cells can be activated [36]. Therefore, cell attachment, morphology and viability are considered important indicators of the cytocompatibility of biomaterials [37]. In addition, the toxicity of the biomaterials’ surface can lead to an inflammation reaction and affect the stability and long-term outcome of the biomaterial [38]. 

Several studies reported that carbon pollution on the surfaces of titanium- and zirconia-based dental implant materials was reduced following UV treatment, and the amount of hydroxyl groups was increased, leading to a hydrophilization of the surfaces and improving the interactions between biomaterials and cells [39,40]. Furthermore, UV irradiation can transform the electrostatic state of the surfaces of materials into a positive charge, which may enhance protein adsorption and cellular adhesion [41]. UV light is able to enhance the osteoconductive capacity of biomaterials [42]. In the present study, the attachment and adhesion of HGFs and L929 cells was improved on surfaces of titanium, zirconia and BioHPP following a short of UV light-treatment of 12 min, which may also be related to the formation of superhydrophilicity and a positive charge, and the reduction in carbon remnants. The work of Yang et al. found that 24 h of UV light treatment had a positive effect on the behavior of HGFs on zirconia, including proliferation, cell adhesion, and collagen release, which is consistent with the results after only 12 min of treatment in this study, that is, by far, easier to integrate into daily clinical routine [43]. In addition, compared to non-treated controls, UV light significantly increased the viability of L929 and HGFs on the surfaces of BioHPP, and decreased the toxicity of L929 on the surface of titanium and zirconia. These results support the suggestion that short-time UV light-treatment may be an effective method to increase the compatibility of soft-tissue cells. 

NTP is also able to form hydroxyl groups on the surfaces of biomaterials by inducing a one-electron oxidation of atmospheric water, leading to a hydrogen ion and a hydroxyl radical [44]. Comparable to UV light, hydroxyl groups can increase hydrophilicity, as well as chemical interactions between cells and surfaces of biomaterials [18]. NTP may also be able to increase immunogenic cell death by using oxidative stress pathways, in order to promote cancer immunotherapy [45]. Recent studies even describe a growth-promoting effect in chickens, after exposing NTP to fertilized eggs in vivo [46]. After 1 year of observation, NTP did not induce negative side effects like chronic inflammation or tumor formation in mice that received a full-thickness ear wound and were treated with NTP [47]. Low-temperature atmospheric pressure plasma has proven to be effective in reducing the formation of bacterial colonies in vitro and could have a positive effect in the treatment of medication-related osteonecrosis of the jaws [48]. The work of Daeschlein et al. found that two different plasma devices were able to erase eight different mycobacterial species effectively in vitro [49]. Additionally, Kang et al. found that NTP treatment improved cell migration and collagen production of fibroblasts derived from human skin, by increasing the expression of EGFR, STAT3, and Type I collagen, which might also be a reason for the improved attachment and adhesion of L929 and HGFs on NTP-treated BioHPP after 2, 24 and 48 h [50]. The significant increase in L929 and HGF viability on BioHPP, following plasma treatment after 48 h of incubation, compared to non-treated surfaces is further proof that NTP can increase the biocompatibility of BioHPP. Generally, the number of attached cells and cell processes of L929 on titanium and zirconia surfaces were higher in the oxygen plasma treatment group than in the argon plasma treatment group, but both were increased compared to controls. However, attachment and cell extensions were not increased for argon plasma-treated HGFs on both surfaces. These results suggest that oxygen plasma treatments can improve the early attachments of fibroblasts. In a recent work, Zheng and colleagues found similar results after treating zirconia surfaces using argon plasma, helium plasma, or a mixture of argon and oxygen plasma, and culturing HGFs on them [51]. Generally, cells spread better on plasma-treated surfaces and had more cell protrusions, but the density of cells increased most on the surfaces that were treated with the mixture of argon and oxygen plasma. The work of Lee et al. found that the early attachment of HGFs on titanium disks after 4 h of incubation, following treatment by an atmospheric plasma jet and a treatment interval of 10 s using normal air, can be improved [52]. 

However, the effect of argon plasma treatment on the two different types of cells (L929 and HGFs) in this study was different. Compared to non-treated surfaces, the viability of HGFs was decreased after argon plasma treatment in the titanium and zirconia groups, which indicates that oxygen plasma may be preferable to argon plasma in the treatment of these surfaces when used as abutment materials. In previous studies, the viability of murine osteoblast-like cells MC3T3-E1 was significantly increased on argon plasma-treated micro-rough surfaces of titanium disks after 48 h of incubation, which is comparable with the effect of this surface treatment on L929 cells in this study, but contrary to its effect on HGFs [16]. Therefore, different cellular reactivity to different carrier gases may be related to the source of the cells and the type of material being processed, but the underlying mechanism still requires further investigation. 

The results of this study suggest that both UV light- and oxygen plasma-treatment may improve the attachment of soft-tissue cells, in order to establish a fast and dense peri-implant soft tissue seal on titanium, zirconia and BioHPP implant abutments. However, the effects of argon plasma-treated surfaces on the cytocompatibility of soft-tissue cells varied with the type of cells and the treated materials. Additional studies should be performed to evaluate the identified effects on cell attachment and viability.

## 4. Materials and Methods 

### 4.1. Sample Preparation and Surface Characterization

Titanium disks were made from titanium grade 4 (15 mm in diameter, 1.5 mm in thickness; Camlog, Basel, Switzerland), zirconia disks were made from tetragonal zirconia polycrystal (ZrO_2_ 95%, Y_2_O_3_ 5%, 15 mm in diameter, 1.5 mm in thickness; Camlog, Basel, Switzerland) and PEEK disks were made from high-performance polyetheretherketone strengthened by 25% ceramic particles (BioHPP^®^, 15 mm in diameter, 1.5 mm in thickness; bredent GmbH, Senden, Germany). The surfaces of all titanium, zirconia and BioHPP samples were polished, sterilized and stored in customary packages for at least 4 weeks. Sample roughness was analyzed by confocal microscopy, according to ISO 4288:1996. The mean arithmetic roughness (Ra) of titanium, zirconia and BioHPP samples was 1.8 μm, 1.4 μm and 1.5 μm, respectively. 

### 4.2. UV Light and NTP Treatment

Titanium, zirconia and BioHPP samples were randomly divided into one group of non-treated samples (controls) and three experimental groups. Disks in the first of the experimental groups were treated with UV light, using an UV light oven, which generated UV light with an intensity of 0.15 mW/cm^2^ (λ = 253.7 nm). The disks in the other two groups were either treated with argon plasma or oxygen plasma, using an NTP reactor (generator frequency 100 kHz, input power 24 W, system pressure 1 mbar, gas flow rate 1.25 sccm, and gas purity >99.5% Diener Electronic GmbH, Ebhausen, Germany). All samples in the experimental groups were treated for 12 min.

### 4.3. Murine Fibroblast L929 and HGF Cell Culture

For all experiments, L929 murine fibroblast cells (C57BL/6, Sigma–Aldrich, Munich, Germany) and human gingiva fibroblast cells (HGF, ATCC, Wesel, Germany) were used. L929 were cultured in minima essential medium (MEM, Gibco, Invitrogen, Paisley, UK) supplemented with 10% fetal bovine serum (FBS, Gibco, Invitrogen, Paisley, UK) and 1% penicillin/streptomycin (P/S, Gibco, Invitrogen, Paisley, UK). HGFs were cultured in Dulbecco’s modified Eagle’s medium (DMEM; Gibco, Invitrogen, Paisley, UK) supplemented with 10% FBS and 1% P/S. Cells were incubated in a humified atmosphere of 95% air and 5% CO_2_ at 37 °C. Cells were detached at 80% confluence using 0.05% trypsin with ethylenediaminetetraacetic acid (Gibco, Invitrogen, Paisley, UK) and counted in a hemocytometer (Hecht Assistant, Sondheim vor der Rhon, Germany). Cells were seeded onto the treated or non-treated disks at a density of 0.5 × 10^5^ /cm^2^, assessing cell attachment and morphology, and 1 × 10^5^ /cm^2^ assessing viability and cytotoxicity.

### 4.4. Cell Attachment and Morphology

Confocal laser scanning microscopy (TCS SP8 X, Leica Microsystems, Wetzlar, Germany) was used to assess cell attachment and morphology using a 60-fold objective lens. After 2 and 24 h, cells were fixed by 4% paraformaldehyde for 30 min, and permeabilized with 0.1% Triton X-100/PBS (Gibco, Invitrogen, Paisley, UK) for 15 min at room temperature. After rinsing three times using PBS, F-actin filaments were stained using a fluorescent dye (biotinylated phalloidin, Alexa Fluor 488 green, 1:1000; Thermo Fisher Scientific, Waltham, MA, USA) and incubated for 60 min at room temperature. After that, samples were washed with PBS three times and dried in normal air. Antifade Mountant (Fluoromount-G, Southern Biotech, Birmingham, AL, USA) was used to fix all samples on glass bottom dishes (WillCo-Dish, Amsterdam, The Netherlands) and stored in the dark at 4 °C. 

The number of living cells attached to the surfaces of the disks was assessed using fluorescence microscopy (Eclipse E200, Nikon, Tokyo, Japan) after 2, 24 and 48 h of incubation following live–dead staining (LDS) using fluorescin diacetate/propium iodide (FAD/PI, Sigma-Aldrich, Munich, Germany). The number of living cells attached to the disks were measured by image J software (release 1.5 h, U.S. National Institutes of Health, Bethesda, MD, USA). Three images were used to measure the number of cells in each group.

### 4.5. Viability Assay

Viability was assessed using CellTiter-Blue viability assay (Promega, Fitchburg, WI, USA). After 48 h of incubation, 60 μL/well of CellTiter-Blue reagent was added and cells were incubated again in standard cell culture conditions for another 4 h. An amount of 100 μL of the culture supernatant was transferred to a 96 well-plate and fluorescence was measured using a multi-well spectrophotometer (ELISA reader), at an excitation wavelength of 560 nm and an emission wavelength of 580 nm. Values in each experimental group were normalized against the values of controls.

### 4.6. Cytotoxicity Assay

Cytotoxicity was assessed by a lactate dehydrogenase cytotoxicity assay (LDH-Cytotoxicity Assay Kit II, BioVision, Milpitas, CA, USA). After 48 h of incubation, 10 μL of the culture supernatant was incubated with 100 μL LDH reaction mix for 30 min. The absorbance was measured using a multi-well spectrophotometer (ELISA reader) equipped with 450 nm and 690 nm (reference wavelength) filters. RM-A, a polyurethane film containing 0.1% zinc diethyldithiocarbamate (Hatano Research Institute, Food and Drug Safety Center, Hadano, Kanagawa, Japan), was used as positive control reference material.

### 4.7. Statistical Analysis

Statistical analysis was performed using SPSS 21 (IBM, Armonk, NY, USA). The normality of viability and toxicity values were assessed using the skewness–kurtosis method. Afterwards, data were analyzed using a one-way analysis of variance (ANOVA) in cases of normal distribution. For skewed data, non-parametric Kruskal–Wallis tests were used. For all results, statistical significance was set at *p* < 0.05.

## 5. Conclusions

UV light and oxygen plasma treatments may improve the attachment, proliferation and viability of soft tissue cells on machined titanium, zirconia and ceramic reinforced PEEK surfaces. In the present study, argon plasma treatment showed only minor effects on the cytocompatibility of soft tissue cells.

## Abbreviations

HGFs	Human gingival fibroblast
NTP	Non-thermal plasma
PEEK	Polyetheretherketone
UV	Ultraviolet
